# HIC-5: A Mobile Molecular Scaffold Regulating the Anchorage Dependence of Cell Growth

**DOI:** 10.1155/2012/426138

**Published:** 2011-11-17

**Authors:** Motoko Shibanuma, Kazunori Mori, Kiyoshi Nose

**Affiliations:** Department of Cancer Cell Biology, Showa University School of Pharmacy, 1-5-8 Hatanodai, Shinagawa-ku, Tokyo 142-8555, Japan

## Abstract

HIC-5 is a multidomain LIM protein homologous to paxillin that serves as a molecular scaffold at focal adhesions and in the nucleus. It forms mobile molecular units with LIM-only proteins, PINCH, and CRP2 and translocates in and out of the nucleus via a nuclear export signal (NES). Of note, NES of HIC-5 is distinctive in its sensitivity to the cellular redox state. Recently, the mobile units of HIC-5 have been suggested to be involved in the regulation of the anchorage dependence of cell growth. On loss of adhesion, an increase in reactive oxygen species in the cells modifies NES and stops shuttling, which leads to cell-cycle control. More specifically, the system circumvents nuclear localization of cyclin D1 and transactivates p21^Cip1^ in detached cells, thereby avoiding anchorage-independent cell growth. Thus, the HIC-5-LIM only protein complex has emerged as a fail-safe system for regulating the anchorage dependence of cell growth.

## 1. Introduction

**Figure 1:**
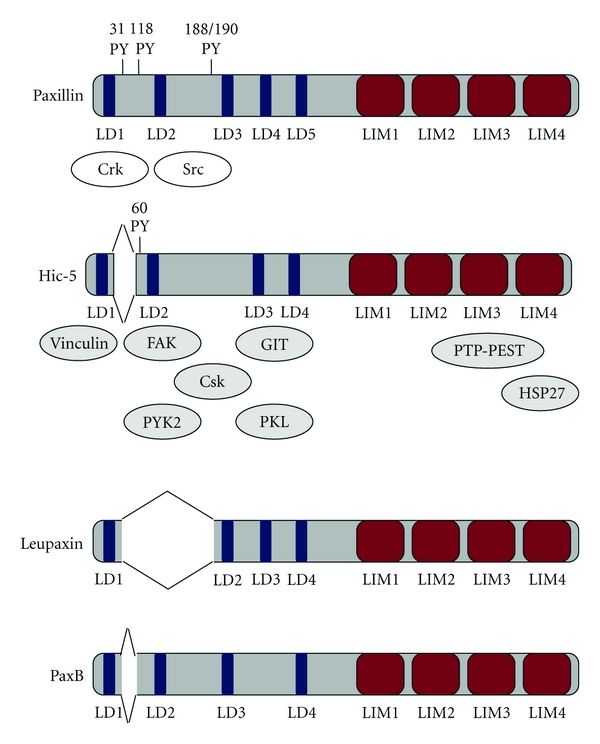
The paxillin/focal adhesion-associated adaptor protein family; domain structure and binding factors. The paxillin family includes HIC-5, Leupaxin, which is preferentially expressed in hematopoietic cells, and PaxB, an orthologue of paxillin in the slime mould *Dictyostellium discoidium*. The family members share many of their structural characteristics and binding factors. They have four to five leucine-rich motifs (LD repeats) in the N-terminal half and four cysteine-rich regions composed of two zinc fingers (LIM domains) in the C-terminal half. These domains mediate the protein-protein interactions that allow paxillin to bind a variety of structural and signalling molecules, such as the structural protein vinculin, the SH2-SH3 adaptor protein Crk, Src, focal adhesion kinase (FAK), PTK2B protein tyrosine kinase 2 beta (PYK2), a negative regulator of Src, the Csk nonreceptor tyrosine kinase, the G protein-coupled receptor kinase interactor Arf GAP1 (GIT-1), paxillin-kinase linker (PKL), protein tyrosine phosphatase-PEST (PTP-PEST), and heat shock protein 27 (HSP27). The LIM domains also mediate the localization of Hic-5 at the nucleus and at focal adhesions. HIC-5 has the same binding partners, except for Crk and Src, as paxillin.

Hydrogen peroxide-inducible clone 5 or *Hic-5* is a gene we isolated by subtractive hybridization in 1994 as a cDNA clone induced by transforming growth factor *β* (TGF-*β*) or hydrogen peroxide [1]. At that time, we studied TGF-*β* signalling and pursued the possibility that reactive oxygen species (ROS) function was an intracellular TGF-*β* signal. After isolating the gene, we conducted a number of studies of *Hic-5* at a molecular as well as cellular level. Its amino acid sequence revealed that HIC-5 is a homologue of paxillin, which is a multidomain LIM (Lin-11, Isl-1, and Mec-3) protein that is localized at focal adhesions and was originally identified as a substrate of the *v-src* oncogene [2] (Figure 1). Together with its family members (Leupaxin specifically expressed in lymphocytes, PaxB, an orthologue of paxillin in slime mold, and HIC-5), paxillin has now been established as a molecular adaptor that transduces signals in response to changes in the adhesion environment of cells. A famous example of a molecular adaptor is the Grb2-SOS system that transduces signals from growth factor receptors to RAS. Paxillin transduces signals from extracellular matrix receptors, integrins, to intracellular downstream molecules such as MAP kinase. 

Of these family members, HIC-5 is most homologous to paxillin, and thus, analyses of HIC-5 have been conducted in reference to and in comparison with paxillin. For example, the intracellular localization of HIC-5 is, like paxillin, mainly confined to so-called focal adhesion sites where cells adhere to the extracellular matrix via integrins. In terms of expression in tissues and cell types, paxillin is relatively ubiquitously expressed, whereas expression of HIC-5 is prominent in the smooth muscle layer of tissues such as the large intestine and uterus [3]. Furthermore, expression of HIC-5 is relatively high in the lung and spleen [1]. In cell culture systems, HIC-5 expression is detectable in most cell lines with varying degrees of expression. High expression of HIC-5 is detected in mesenchymal cell lines including fibroblastic and osteoblastic cell lines; however, it is generally low in epithelial cell lines. In a knockout mouse model, HIC-5 was suggested to be inessential for the development and maintenance of homeostasis of the animal, and no remarkable functional abnormality was found under standard rearing conditions [4]. In contrast, the paxillin knockout mouse is reportedly embryonic lethal [5]. Similar to fibronectin, it exhibits abnormal development of extraembryonic tissues and heart and body segmentation, resulting in death at 9.5 foetal days. The embryonic lethality of the paxillin knockout mouse means that HIC-5 cannot substitute the functions of paxillin, at least those associated with development. These results together with the abovementioned differences in expression patterns indicate that it is most likely that paxillin and HIC-5 have different functions in mammals.

## 2. Structure of HIC-5 and Interacting Factors 

The genomic structure of *Hic-5* features a long intron between the N-terminal and C-terminal domains, a sign that *Hic-5* evolved from the fusion of two different genes [6]. Accordingly, the protein structure can also be broadly divided from the centre into N-terminal and C-terminal regions.

 The N-terminal region comprises four domains, the LD domains, which are rich in Leu and Asp; LD1 is deleted in one isoform. The C-terminal region comprises four LIM domains having two zinc fingers (Figure 1). These features are almost identical to those of paxillin, with minor differences in the number of LD domains in the N-terminal region (five for paxillin and four for HIC-5). Given that both the LD and LIM domains are protein-protein interacting domains, it is naturally assumed that paxillin family members are adaptor molecules that provide multiple proteins with interfaces to facilitate their interaction and cooperation.

 Based on these structural features, further analyses have successfully identified a number of interacting factors. In particular, in the case of adaptor molecules, identification of their interacting factors is crucial for inferring their functions. Several interacting factors of paxillin have been identified previously. Therefore, we began to study their interaction with HIC-5. Isolation of new interacting factors was also attempted, and we found an array of proteins that interact with each of the LD and LIM domains of HIC-5 [2] (Figure 1). Consistent with the localization of HIC-5 at focal adhesions, most of the factors identified are those contained in the cytoplasmic domain complex of integrins (discussed below) and are involved in integrin signal transduction and/or in the control of actin cytoskeleton dynamics. More specifically, the factors include signalling molecules such as protein tyrosine kinase 2 beta (PYK2), *c-src* tyrosine kinase (Csk), and focal adhesion kinase (FAK) [7, 8]. Using a yeast two-hybrid method with the N-terminal and C-terminal domains of HIC-5 as bait, we originally characterized G protein-coupled receptor kinase interacting Arf GAP1 (GIT-1) as an LD3 interacting factor [9] and protein tyrosine phosphatase, nonreceptor type 12 (PTP-PEST) as an LIM3 interacting factor [10]. Vinculin and talin are structural proteins involved in the architecture of focal adhesions and the actin cytoskeleton, and these also interact with HIC-5 [8].

 Most of the factors that bind to HIC-5 also interact with paxillin although paxillin additionally binds Src and Crk (*v-crk* sarcoma virus CT10 oncogene homologue), which do not bind to HIC-5 [2, 8]. Accordingly, HIC-5 and paxillin hypothetically compete for these interacting factors. This difference is presumably because of the presence of tyrosine residues (Y31, Y118, Y188, and Y190) in paxillin, which do not exist in HIC-5 although no definitive answer has been obtained yet. Furthermore, it is known that paxillin binds the cytoplasmic domains of *α*4, *α*9, and *β*3 integrins [11], and HIC-5 interacts with the domain of *α*4. No particular pattern of interaction has been identified for the LD and LIM domains of HIC-5 and paxillin and their corresponding interacting factors. Thus, the study as above identified only factors whose localization and functions are related to focal adhesions. Furthermore, no factors that interact specifically with HIC-5 were identified.

HIC-5 has also been found to be localized in actin stress fibers, while paxillin is not [3]. Here, HIC-5 appeared to interact with cysteine-rich protein (CRP) 2, which belongs to the LIM-only group of proteins [3]. This finding prompted us to search for other LIM-only proteins that interact with HIC-5, and we identified a particularly interesting new cysteine and histidine-rich protein (PINCH) that binds HIC-5 [12]. Further investigation demonstrated that LIM4 in the C-terminal is important for HIC-5 interaction with these LIM-only proteins. In other words, the fourth LIM domain located at the endmost C-terminal region has a unique property, whereby it interacts with the same LIM domain, thus enabling HIC-5 to form LIM-LIM homo- and hetero-oligomers [12]. Consequently, HIC-5 exists as homo-oligomers in cells, which appear to be important for HIC-5 localization in the nuclear matrix (unpublished data). Hetero-oligomer formation between HIC-5 and PINCH/CRP2 is essential for the two LIM-only proteins to localize at particular sites in cells [12]. This means that HIC-5 directs their subcellular localization (as discussed below). It should be noted that LIM4 of paxillin cannot form oligomers and does not interact with PINCH or CRP2. This difference is surprising given the high structural homology between HIC-5 and paxillin. Another molecular difference is that paxillin is highly tyrosine-phosphorylated during adhesion and growth signalling, whereas HIC-5 is not generally tyrosine-phosphorylated to a significant level. This might be explained by the fact that HIC-5 has no tyrosine residues *per se* to be phosphorylated [13]. Thus, unlike paxillin, HIC-5 appears to not have a function in adhesion and growth signalling based on tyrosine phosphorylation although in human platelets, HIC-5 has been shown to be tyrosine-phosphorylated with aggregation. 

Studies at a molecular level have characterized paxillin and HIC-5 as being tyrosine-phosphorylated and forming LIM-LIM oligomers, respectively, which possibly underlies the functional difference between them. Paxillin may transduce adhesion/growth signalling, and HIC-5 organizes a fail-safe system for the adhesion dependence of cell growth, as discussed below.

## 3. Functions of HIC-5 at Focal Adhesions and in the Nucleus

Adhesion between cells and the extracellular matrix is achieved by integrins, a group of transmembrane proteins that act as receptors for the extracellular matrix. The resultant adhesion of cells to the extracellular matrix, in turn, promotes aggregation between integrins, resulting in the formation of a protein complex known as a focal adhesion at the site of adhesion. Importantly, a focal adhesion is not just a physical structure but also a molecular apparatus that senses the adhesion status between cells and the extracellular matrix and sends signals to the inside of cells, thereby coordinating their motility, survival, and proliferation in response to adhesion status [14, 15]. During signalling by integrins whose cytoplasmic domain is very short and contains no enzymatic catalytic domain, multiple proteins assemble at the cytoplasmic domain through protein-protein interactions, forming an undercoat structure. These proteins include several enzymes such as FAK and SRC, through which the signal is emitted to the inside of the cell [14, 15].

 HIC-5 and paxillin act as adaptor molecules in this integrin undercoat complex and play roles in controlling enzyme activity by mediating the above-mentioned interactions between binding factors, thereby participating in the regulation of integrin signalling. A detailed study has shown that HIC-5 plays a role in negatively regulating FAK by competing with paxillin [16]. However, under normal culture conditions, cellular behaviours including growth and motility are hardly affected by HIC-5 expression in most cases. Therefore, HIC-5 appears to not be required for any particular function at focal adhesions under normal cellular conditions so long as the adhesion status is maintained. Normal phenotype observed in the knockout mice strongly supports this idea. Rather, HIC-5 may have a critical function in suppressing excess changes in adhesion and the cytoskeleton structure by antagonizing paxillin when the maintenance of healthy adhesion is in jeopardy under stressful conditions affecting adhesion [3, 16]. In sharp contrast, abnormal cell motility and spreading is observed under normal conditions in paxillin^−/−^ cells in which the focal adhesions are disorganized and integrin signalling by FAK and the downstream MAP kinase is disturbed [5].

 In parallel with localization at focal adhesions, HIC-5 simultaneously shuttles between focal adhesions and the nucleus, as described below. This fact lead us to investigate the possible function of HIC-5 in the nucleus, and a series of study demonstrated that HIC-5 was capable of regulating expression of the *c-fos* and p21^Cip1^ genes [17, 18]. This involvement in transcription was not observed with paxillin. Further study demonstrated that HIC-5 promotes the formation of a transcriptional complex, specifically with the transcriptional coactivator p300 and transcription factors Sp1 and Smad3, on DNA [18]. In other words, HIC-5 appears to function as an adaptor for transcription-associated factors in the nucleus by acting as a scaffold for transcriptional complex formation, as is the case for integrin signalling at focal adhesions. A similar function of HIC-5 in the nucleus has been suggested by other researchers who recloned HIC-5 as an androgen receptor coactivator 55 kDa protein (ARA55), in 1999 [19, 20]. It has also been reported that HIC-5/ARA55 is a coactivator of PPAR*γ* and others [21, 22]. Taken together, these results indicate that HIC-5/ARA55 may serve as a scaffold for a fairly wide range of transcriptional activities in the nucleus.

In summary, a growing body of evidence suggests that the function of HIC-5 in the nucleus is to facilitate transcription complex formation by interactions with several transcription-associated factors. However, taking into consideration the fact that under normal conditions, HIC-5 is mainly localized at focal adhesions and not in the nucleus at a detectable level, we should be cautious about this conclusion. Rather, we postulate that HIC-5 has some roles in the nucleus, primarily under conditions where focal adhesions have been disrupted and that localization of HIC-5 in the nucleus then becomes discernable.

## 4. Nuclear-Cytoplasmic Shuttling of HIC-5 and Its Biological Significance: A Fail-Safe System for Anchorage Dependence of Cell Proliferation

In 2003, we found that HIC-5 shuttles between focal adhesions and the nucleus [23]. Our initial observations indicated that HIC-5 changed its localization to the nucleus following treatment with hydrogen peroxide, suggesting that besides focal adhesions, HIC-5 can also localize in the nucleus based on the cellular conditions. Finally, we found that HIC-5 shuttles between focal adhesions and the nucleus constantly in normal adhesion cells and that it does so in company with PINCH and CRP2, its LIM-LIM hetero-oligomer partners.

 This molecular-level analysis of the shuttling ability of HIC-5 identified NES around the LD3 domain [23]. A nuclear localization signal is carried by four LIM domains in the C-terminal region. Of note, there are specific cysteine residues upstream of the NES consensus sequence in HIC-5 that enable NES to sense the intracellular redox state. Because of this, NES is interrupted to stop the export of HIC-5 from the nucleus under conditions such as hydrogen peroxide treatment, which results in the accumulation of the protein in the nucleus [23]. In short, HIC-5 shuttles between focal adhesions and the nucleus in normal situations, but it accumulates and becomes functional in the nucleus when the intracellular redox state becomes more oxidised. Of interest, when cells are deprived of adhesion to a substrate, the ROS concentration in the cells increases [24].

 These findings indicate that HIC-5 is a shuttle protein and an ROS effector, just as the name suggests. Most ROS effectors are signalling molecules centred on tyrosine phosphatase or transcription factors. HIC-5 is probably the first ROS effector to act as an adaptor. In addition, HIC-5 is unique in that it uses ROS as a switch for changing its localization by sensing ROS in NES. A group of LIM proteins including paxillin, zyxin, LLP, and a thyroid hormone receptor-binding factor, Trip 6, has also been shown to communicate between focal adhesions and the nucleus although the controlling mechanisms and significance of this shuttling are poorly understood [25].

 Next, we explored the significance of the shuttling of HIC-5 between the two compartments. Hypothetically, by shuttling, HIC-5 connects the adhesion status with nuclear activity. We focused on the adhesion (anchorage) dependence of cell proliferation as an example of a phenomenon that requires such a coupling mechanism.

 Numerous studies have defined the roles of adhesion signals mediated by integrin-extracellular matrix (ECM) interaction in cell-cycle progression, Basically, integrin-ECM-mediated signaling potentiates and prolongs the growth factor receptor-mediated mitogenic signalling and is required from mid- to late-G_1_ phase in various events associated with cell-cycle progression, such as upregulation of G_1_-phase cyclin-dependent kinase (CDK) activity, Cip/Kips downregulation, association of cyclin E with CDK2, pRB phosphorylation, and cyclin A expression. As a result, loss of adhesion generally causes complete G_1_ phase cell-cycle arrest in nontransformed cells; moreover, in susceptible cells, it leads to anoikis, a specific type of apoptosis caused by the detachment of a cell from its supportive matrix, which was first described in epithelial and endothelial cells [26, 27]. In contrast, transformed cells usually circumvent the anchorage requirement in cell-cycle progression. Their anchorage-independent survival and growth is well known as a hallmark of cellular transformation and correlates with tumorigenicity *in vivo*. Mechanistically, the anchorage-independent growth is considered to be based on an abnormal activation of the G_1_-phase cyclin CDKs uncoupled from anchorage. In general, an oncogenic pathway activates a robust and/or constitutive mitogenic signal, which is presumed to reduce the requirement for integrin-ECM-mediated signalling and its importance as a booster of growth factor receptor-mediated mitogenic signalling in the transformed cells. Among the downstream pathways of oncogenic signals, the activation of the phosphatidylinositol 3-kinase/Akt pathway is crucial for the induction of anchorage-independent growth and cell survival.

Among the events required for the cell-cycle progression, activation of CDK4/6 by cyclin D is one of the most important events, because it promotes the G_1_/S transition; that is, it serves as a determinant of cell-cycle progression along with cyclin E/CDK2 activity. Importantly, activation of this complex is sensitive to adhesion status as well as growth stimuli, and it plays a role in coupling adhesion status with cell-cycle progression. Thus, under conditions of inappropriate adhesion, it operates by circumventing progression of the cell cycle. In other words, besides acting as an engine of cell-cycle progression, CDK4/6-cyclin D functions as a security system for the anchorage dependence of cell proliferation. Mechanistically, the expression level of cyclin D is regulated not only by growth signals but also by adhesion signals, and an insufficiency of either signal type decreases its expression [28, 29]. The level of expression of other cyclins rather automatically cycles up and down with progression of the cell cycle, as their name suggests. Thus, Cyclin D is a key molecule in regulation of the cell cycle, and abnormal cyclin D behaviour is directly linked to aberrant proliferation of cells, which is supported by the fact that cyclin D is a proto-oncogene. 

Accordingly, not only the amount of cyclin D in cells but also its subcellular localization is rigorously regulated by signalling, and cyclin D localizes in the nucleus only during G_1_/S phase, when nuclear localization is essential for its function; it is exported from the nucleus during the other phases [30]. When this does not occur, it becomes oncogenic, as has been shown by observations, where the nuclear localization of cyclin D promoted cell proliferation; its oncogenic potential is closely related to its nuclear localization ability [31]. Regulation of the nuclear localization of cyclin D is so important that we hypothesized that it may also be regulated by adhesion status. Therefore, we examined the relationship between the nuclear localization of cyclin D1 and the adhesion status of cells. In adherent cells at G_1_ phase, cyclin D1 is localized in the nucleus. However, this localization changes immediately to the cytoplasm when cells are suspended [24]. This result supports the above hypothesis that the nuclear localization of cyclin D1 is also regulated by the adhesion status. This regulation would be of great help in avoiding unacceptable cell growth when cells are detached; most interestingly, HIC-5 is deeply involved in this mechanism. Basically, HIC-5 and cyclin D1 translocate between the cytoplasm and nucleus through the same CRM 1-dependent nuclear export system, and for this reason, they compete for this export system. The presence of PINCH with HIC-5 is important for the competition between HIC-5 and cyclin D1. A critical step is that HIC-5 stops shuttling in cells on loss of adhesion, the consequence of which is the export of cyclin D1 in place of HIC-5 outside the nucleus. The arrest of shuttling in nonadherent cells is caused by an elevated level of ROS, which inactivates NES [24].

**Figure 2:**
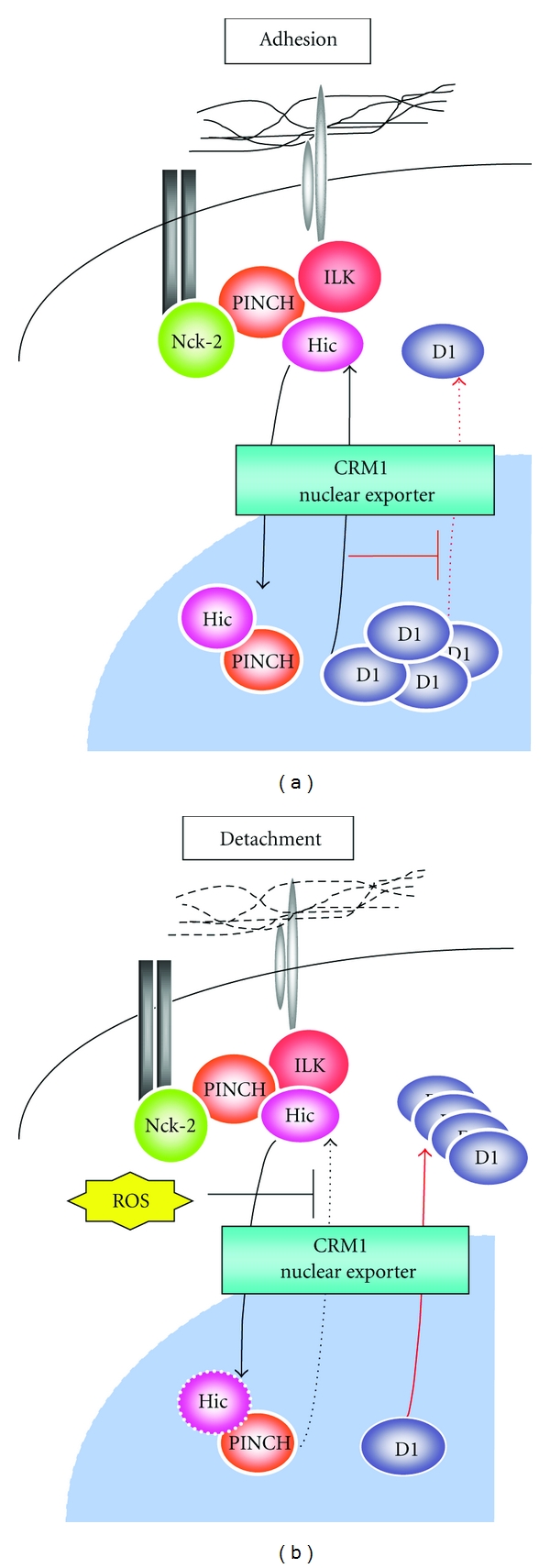
A mobile HIC-5/PINCH scaffold regulating cyclin D1 subcellular localization in response to adhesion status. Cyclin D1 is retained in the nucleus in adherent cells such as mouse primary fibroblasts, C3H10T1/2 fibroblasts, and mammary epithelial NMuMG cells by the CRM1-dependent nuclear export of HIC-5 with the aid of PINCH, which counteracts the nuclear export of cyclin D1. The higher affinity of HIC-5 for CRM1 favours the export of HIC-5. In detached cells, the nuclear export of HIC-5 is inhibited by elevated levels of ROS, and cyclin D1 is actively transported out of the nucleus, which results in a decrease in its nuclear localization (see further details in [24]).

To summarize, the biological significance of the shuttling of HIC-5 is competitive localization of cyclin D1 in the nucleus in adherent cells with the aid of PINCH. Importantly, the shuttling of HIC-5 is in the off state in nonadherent cells. This results in critical acceleration of the export of cyclin D1 to the cytoplasm and in the arrest of cell proliferation under abnormal conditions. This mechanism, which is regulated by the on/off status of HIC-5 shuttling, functions as a fail-safe system for ensuring the anchorage dependence of cell proliferation (Figure 2).

Most recently, we found an additional mechanism involving HIC-5 for inducing growth arrest in detached cells. The mechanism targets p21^Cip1^, a cyclin-dependent kinase inhibitor that is famous as the downstream target of the tumour-suppressor gene p53. This gene is well known to be transactivated by p53 in response to a variety of stresses and to stop G_1_/S progression by inhibition of cyclin D-CDK4/6 or cyclin E-CDK2. Of note, when cells are deprived of adhesion, p21^Cip1^ is upregulated and plays a critical role in growth arrest [29]. Our recent investigation suggests that HIC-5 participates in the transactivation of p21^Cip1^ in response to loss of anchorage. In this case, HIC-5 cooperates with CRP2, another LIM-only protein shuttling partner, to transactivate the KLF4 and Runx1 sites upstream of the gene (unpublished data).

**Figure 3:**
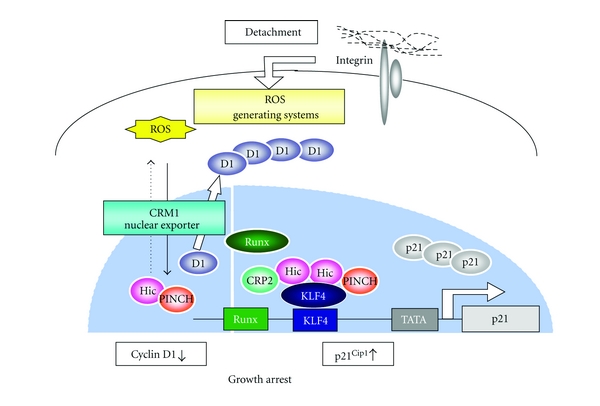
A fail-safe system where HIC-5 ensures the anchorage dependence of cell growth through LIM-LIM interactions. On loss of adhesion, HIC-5 activates two modes of a fail-safe system in collaboration with PINCH and CRP2 to prevent anchorage-independent cell growth. The HIC-5-PINCH unit halts its nuclear-cytoplasmic shuttling and makes way for cyclin D1 to be exported from the nucleus by CRM1, as illustrated in Figure 2, while simultaneously transactivating p21^Cip1^ in the nucleus together with another HIC-5 unit, HIC-5-CRP2. As such, the dual systems, which target cyclin D1 and p21^Cip1^, respectively, cooperatively stop detached cells from proliferating and thus avoid unacceptable anchorage-independent growth (see further details in [24]).

 In conclusion, HIC-5 has emerged as a critically important molecule for regulation of the anchorage dependence of cell proliferation. More specifically, HIC-5 organizes two types of mobile LIM-LIM oligomer platforms with PINCH and CRP2, which collaboratively prevent anchorage-independent cell growth. For this purpose, HIC-5/PINCH regulates the nuclear localization of cyclin D and HIC-5/CRP2 is operational in the transactivation of p21^Cip1^ (Figure 3). 

## 5. Adhesion Status of Cells and HIC-5

A series of studies by us and others have suggested interesting possibilities for the function of HIC-5 at focal adhesions and in the nucleus. We also have originally addressed its role as a mobile scaffold for the anchorage dependence of cell growth. However, in terms of an active role of HIC-5 under normal adhesion conditions, the evidence presented thus far is not fully convincing for either localization. We speculate that under normal adhesion conditions, HIC-5 senses the adhesion status based on the fact that its NES is protected from attack by ROS in the adhesion complex and is able to assist HIC-5 shuttling. Once the cells lapse into a state of abnormal adhesion, such as detachment from the matrix, the adhesion structure becomes disrupted along with ROS production; under such conditions, NES of HIC-5 is unmasked, becomes exposed to ROS, and is modified to stop the shuttling. When shuttling of HIC-5 is stopped, cell growth is arrested through the mechanisms involving cyclin D and p21^Cip1^. In other words, HIC-5 organizes a fail-safe system that ensures the anchorage dependence of cell growth. The activation mechanism of this system is oxidative modification of NES of HIC-5 in response to increased production of ROS as well as the collapse of the focal adhesion complex. At this point, it should be noted that the interaction between FAK and HIC-5 is also sensitive to ROS. This is because FAK and HIC-5 interact through LD3 of HIC-5, which contains NES, and this interaction is regulated by the upstream cysteines in the same manner as for NES [32]. Therefore, the interaction between FAK and HIC-5 is also lost under abnormal adhesion conditions, suggesting that the functionality of NES and the complex formation of HIC-5, at least with FAK, in the focal adhesion complex are intimately interrelated.

In an earlier section, we discussed the role of HIC-5 at focal adhesions and on actin stress fibres. Considering that most of these results were observed in cells exposed to stresses such as detachment and stretching but not in cells under normal conditions, HIC-5 functions can, after all, be interpreted as being closely related to the abnormity of the adhesion environment and devoted to suppression of extreme changes in the cytoskeleton as well as to prevention of proliferation under abnormal adhesion conditions.

 A group studying glomerulosclerosis has reported that HIC-5 is significantly induced and involved in the induction of cell death in a pathological model, where mesangial cells were cultured on collagen that was of a different type to the original extracellular matrix [33]. This observation may have captured the essence of HIC-5's function of sensing abnormal adhesion status.

## 6. Cancer Cells and HIC-5

The organization of HIC-5 as a fail-safe system for ensuring the anchorage dependence of cell growth is expected to be involved in the cancer development process. In this point, it should be noted that although HIC-5 is actively involved in the growth arrest of fibroblasts via induction of p21^Cip1^ on loss of anchorage, it is not involved in the anoikis or cell death of epithelial cells (unpublished data). This may be concerned with the fact that p21^Cip1^, which is the target of HIC-5 action and functions in growth arrest, was not induced in normal epithelial cells under detached conditions. Instead, epithelial cells undergo anoikis that is governed by a group of factors specialized for cell death execution. Given these findings together with its relatively high expression in mesenchymal cells, HIC-5 might be an important molecule in the development of sarcoma. Another interesting possibility is that HIC-5 may be involved in the epithelial-mesenchymal transition, as was pointed out by Tumbarello and Turner [34]. The ability of HIC-5 to act as a steroid hormone receptor coactivator has led to studies of its involvement in prostate cancer and endometriosis. Furthermore, a study of a human breast cancer cell line showed that both paxillin and HIC-5 have distinct impacts on cancer cell phenotypes related to metastatic potential. According to this study, in which siRNA was used for knockdown of the genes, both paxillin and HIC-5 function in promoting metastatic potential although through different mechanisms. However, in our recent experiment using shRNA, which would be more adequate for long-term observation than siRNA, we found contradictory results. These studies suggest that HIC-5 is potentially involved in the development of certain carcinomas. However, HIC-5 and cellular transformation remains a controversial issue and awaits further investigation.

In our earlier study, HIC-5 expression dropped sharply and dramatically during immortalization of mouse fibroblasts [35]. In contrast, forced expression of HIC-5 induced a senescence-like phenotype with decreased proliferative potential after repeated passages in some immortalized human fibroblasts [36]. Recently, it was found that the anchorage-independent growth of cancer cells over a long period of time may be negatively regulated by HIC-5 (unpublished data). Thus, the interaction of cell adhesion and the mortality/senescence process would be an interesting subject for future study of HIC-5 biology in association with cancer cells.
